# Synthesis, Development and Characterization of Magnetic Nanomaterials

**DOI:** 10.3390/nano12071036

**Published:** 2022-03-22

**Authors:** Vasileios Tzitzios

**Affiliations:** Institute of Nanoscience and Nanotechnology, National Centre for Scientific Research “Demokritos”, 15310 Athens, Greece; v.tzitzios@inn.demokritos.gr

Magnetic nanomaterials in both thin films and in the form of nanoparticles, with various structures and morphologies, are among the most extensively studied categories of materials. This research interest is mainly due to the growing exploration of new magnetic properties and their use in applied magnetism and the technology of relevant devices, as well as their utilization in commercially available emerging technologies including applications from biomedicine and the environment to data storage and spintronics.

This Special Issue aims to offer readers a compilation of cutting-edge research regarding the synthesis, development and characterization of magnetic nanomaterials, covering a wide spectrum of magnetic nanomaterials and serving as a guide for new students of the field as well as established researchers.

In this Special Issue, there are research articles that focus on the thin film growth of ferromagnetic/semiconducting heterostructures and their relation to structural, magnetic, and transport properties [1,2,3,4], as well as Heusler alloys with ferromagnetic and Weyl semimetals behavior [1,5].

Furthermore, there are research articles that focus on novel organometallic routes, magnetic field nanoparticles assembly [6], heating abilities of magnetic nanoparticles under various shapes and core/shell structure [7,8,9,10].

Papers on the synthesis of magnetic metal–organic frameworks (MOF), and their application in enzymes immobilization are also presented [11].

The results and findings are expected to be useful for researchers who are working in the field of nanomagnetism and nanotechnology. Finally, I would like to express my sincere gratitude to all authors who contributed their innovative research to this Special Issue.

## Data Availability

Not applicable.
